# What's on TV? Detecting age-related neurodegenerative eye disease using eye movement scanpaths

**DOI:** 10.3389/fnagi.2014.00312

**Published:** 2014-11-11

**Authors:** David P. Crabb, Nicholas D. Smith, Haogang Zhu

**Affiliations:** Department of Optometry and Visual Science, School of Health Sciences, City University LondonLondon, UK

**Keywords:** eye movements, scanpaths, glaucoma, perimetry, eye tracking, KPCA, perception, diagnosis procedures

## Abstract

**Purpose:** We test the hypothesis that age-related neurodegenerative eye disease can be detected by examining patterns of eye movement recorded whilst a person naturally watches a movie.

**Methods:** Thirty-two elderly people with healthy vision (median age: 70, interquartile range [IQR] 64–75 years) and 44 patients with a clinical diagnosis of glaucoma (median age: 69, IQR 63–77 years) had standard vision examinations including automated perimetry. Disease severity was measured using a standard clinical measure (visual field mean deviation; MD). All study participants viewed three unmodified TV and film clips on a computer set up incorporating the Eyelink 1000 eyetracker (SR Research, Ontario, Canada). Eye movement scanpaths were plotted using novel methods that first filtered the data and then generated saccade density maps. Maps were then subjected to a feature extraction analysis using kernel principal component analysis (KPCA). Features from the KPCA were then classified using a standard machine based classifier trained and tested by a 10-fold cross validation which was repeated 100 times to estimate the confidence interval (CI) of classification sensitivity and specificity.

**Results:** Patients had a range of disease severity from early to advanced (median [IQR] right eye and left eye MD was −7 [−13 to −5] dB and −9 [−15 to −4] dB, respectively). Average sensitivity for correctly identifying a glaucoma patient at a fixed specificity of 90% was 79% (95% CI: 58–86%). The area under the Receiver Operating Characteristic curve was 0.84 (95% CI: 0.82–0.87).

**Conclusions:** Huge data from scanpaths of eye movements recorded whilst people freely watch TV type films can be processed into maps that contain a signature of vision loss. In this *proof of principle* study we have demonstrated that a group of patients with age-related neurodegenerative eye disease can be reasonably well separated from a group of healthy peers by considering these eye movement signatures alone.

## Introduction

The ever increasing elderly population will cause an “epidemic” of age-related neurological disease in the 21st century. At present, healthcare detection and monitoring of patients with sensory impairments resulting from chronic age-related neurodegenerative disease is done, mainly inadequately, in a clinic; a system that is likely unsustainable in the future. Instead of relying on infrequent tests in a clinic, focus should shift to capturing health-related data acquired as part of a person's ordinary daily activities.

Eye movements are a continuous and ubiquitous part of sensory perception. Whenever we interact with the visual environment we generate saccadic eye movements. Saccades move the eyes in a ballistic fashion from one point to another, interspersed by fixations where the eye is stable. Scanpaths, revealing the sequence of fixations and saccades, collected non-invasively during a period of time that a person is, for example, simply engaged in watching a TV program could give an “*eye movement signature*.” The main idea reported in this paper is to show how these signatures could contain features that can be used to detect if a person has a chronic neurodegenerative condition.

Glaucoma is a generic term for age-related disease of the optic nerve which can lead to irreversible loss of the visual field: the area which can be seen when the eye is directed forward, including both central and peripheral vision. Medical treatment to control the condition is largely successful, but once diagnosed all patients with glaucoma normally need lifelong treatment, and lifelong monitoring within hospitals and clinics, so that any worsening of visual damage can be detected. Therefore, people with glaucoma represent a major workload of eye services, with an estimated one million outpatient appointments per year in the UK for example (National Institute for Health and Clinical Excellence, 2009). The visual field tests (perimetry) are often difficult for patients to do and they are not done with sufficient frequency to adequately monitor most patients (Chauhan et al., 2008; Fung et al., 2013; Glen et al., 2014). Moreover detection rates for the disease are poor with an estimated 50% of all cases undetected (Rudnicka et al., 2006).

In this work we use glaucomatous optic neuropathy as an example of an age-related neurodegenerative disease (Yücel and Gupta, 2008; Caprioli, 2013). The pathogenesis of glaucoma shares many features with other chronic age-related neurodegenerative disease: there is, for example, ample evidence linking the etiology and disease process in glaucoma to Alzheimer's disease (AD) (Bayer et al., 2002; Sivak, 2013). The epidemiology and impact of glaucoma is well known but the pathogenesis of the disease is multifaceted and not well understood. Optic neuropathy is characterized in the clinic by changes in the optic nerve head (ONH) and thinning of the nerve fiber layer. This is almost certainly a result of a non-specific gradual reactive change of glial cells resulting in chronic retinal ganglion cell death and then loss of visual function (Tezel and Fourth ARVO/Pfizer Ophthalmics Research Institute Conference Working Group, 2009; Sivak, 2013).

There are at least two theories to explain eye gaze. In short, eye movement can be driven by factors that purposely direct fixations toward task-driven locations or in the absence of such task demands eyes are likely directed to salient regions. Investigators have already used scanpaths to gain insights into what an observer is doing or their mental state. For example, a large variety of studies have confirmed that eye movements contain rich signatures about an observer. An excellent up-to-date review of the literature is given elsewhere (Borji and Itti, 2014).

Investigators have revealed data showing that simple viewing patterns in controlled experiments can detect eye-movement abnormalities that can discriminate schizophrenia cases from control subjects with good accuracy (Benson et al., 2012). Other workers, extracting salient features from a series of films, have used eye movements to classify patients with attention deficit hyperactivity disorder and Parkinson's disease (Tseng et al., 2013). The work of this type has attempted to demonstrate the classification of clinical populations from natural viewing. Errors in the ability to make anti-saccades (an eye movement purposely directed in the opposite direction from a target) has been repeatedly implicated in AD (Crawford et al., 2013) and patients with AD have also been shown to display irregular eye movements when reading and in other tasks (Lueck et al., 2000; Mosimann et al., 2004). Our laboratory has published preliminary evidence that patients with eye disease make different types of eye movements when compared to age-related control subjects, when performing different types of task (Crabb et al., 2010; Smith et al., 2012; Glen et al., 2013). Other research regarding the nature and consistency of the types of eye movement patterns shown by groups of individuals as they view scenes have been considered (Castelhano and Henderson, 2008; Cristino and Baddeley, 2009; Dorr et al., 2010). Data analyses in these studies typically, and inadequately, rely on simple counts and averages of, for example, number of fixations, saccade amplitude, and region of interest measures. We propose computational approaches to analyze eye-tracking data not used before, considering sequences of saccades within the scanpaths. We also apply machine classifiers to learn combinations of multidimensional features extracted from the scanpaths in order to discover patterns that belong to groups of patients.

Therefore, in this study we test the hypothesis that age-related neurodegenerative eye disease can be detected by examining patterns of eye movement recorded whilst a person naturally watches a TV program. We do this with a case-control study with the aim of providing evidence that patients with a clinical diagnosis of glaucoma can be reasonably well separated from age-related healthy people using data from their scanpaths alone.

## Materials and methods

### Participants

People with glaucoma were recruited from clinics at Moorfields Eye Hospital NHS Foundation Trust, London. All patients had an established clinical diagnosis of chronic open angle glaucoma (COAG) for at least 2 years and were between 50 and 80 years of age. COAG is defined, following clinical guidelines, by the presence of reproducible visual field defects in at least one eye with corresponding damage to the ONH and an open iridocorneal drainage angle on gonioscopy (National Institute for Health and Clinical Excellence, 2009). The diagnosis was made by a glaucoma specialist. A deliberate attempt was made to recruit a sample of patients with a range of disease severity according to visual field loss. Patients were purposely not recruited if they had any ocular disease other than glaucoma (except for an uncomplicated lens replacement cataract surgery). In addition, at the point of recruitment, patients had slit lamp biomicroscopy performed by an ophthalmologist to further exclude any other concomitant macular pathology, ocular surface disease or any significant problems with dry eye.

Healthy people (controls), of a similar age to the patients, were recruited from the City University London Optometry Clinic; this is a primary care center where people routinely receive a full eye examination, which includes measurement of visual acuity, refraction, binocular vision assessment, pupil reactions, slit-lamp assessment of the anterior eye, measurement of intraocular pressure, visual field assessment and indirect ophthalmoscopy of the macula, ONH, and peripheral retina. The City University London Optometry Clinic is located just meters from the hospital meaning all participants were drawn from an identical geodemographic area.

All participants (patients and controls) were required to have a corrected visual acuity of at least 0.18 logMAR (Snellen equivalent 6/9) in each eye at the time of their most recent examination. Astigmatic error was less than ± 2.5 Dioptres in all those recruited. Participants were only recruited if they had no significant health problems (other than their glaucoma for patients), meaning no difficulty with self-care, mobility, pain, anxiety, and depression; this was ascertained at recruitment by self-report to questions based on the EQ-5D instrument (Rabin and de Charro, 2001) added to the participation information sheet. Participants were not enrolled if they were taking any significant medication other than that for their glaucoma. (“Significant medication” included anti-depressants or treatment for diabetes or significant use of β-blocker medication, all of which were deliberately mentioned.) Recruitment of patients and controls was made simultaneously over a period of about 9 months with an effort to make the two groups age-related.

The study was approved by the Moorfields and Whittington Research Ethics Committee, London and the School of Health Sciences Research and Ethics Committee, City University London. Written informed consent, according to the tenets of the Declaration of Helsinki, was obtained prior to examination from each participant. Data was anonymized and stored in a secure database.

### Supplementary vision testing and cognitive screening

All participants underwent vision testing on the day of the study. Visual fields were measured in both eyes with automated perimetry using the Humphrey Field Analyzer (HFA; Carl Zeiss Meditec, CA, USA) employing the central standard 24-2 Swedish Interactive Testing Algorithm. This test is a clinical gold-standard for measuring the severity of functional damage caused by glaucoma. The Glaucoma Hemifield Test (GHT) on the HFA is an established statistical test for early glaucomatous visual field defects (Asman and Heijl, 1992). The GHT was “outside normal limits” for all eyes in all patients and “within normal limits” for all eyes in all controls. If any of the visual fields were flagged by the HFA output as “unreliable,” as assessed by the false positive, false negatives or poor fixation measures, then the test was repeated. The HFA mean deviation (MD) is a standard clinical measure of the overall severity of a visual field defect, relative to healthy age-matched observers, with more negative values indicating greater visual field loss (Flammer, 1986; Artes et al., 2011). MD values were used as a measure of overall glaucomatous disease severity in the patient group. Patients with MD better than −6 dB in both eyes were classified as having early glaucomatous damage whilst those with MD worse than −12 dB in both eyes were considered to have advanced disease; these patients would be typically symptomatic and would, for example, likely fail the visual field component for fitness to drive in the UK (Saunders et al., 2012). All other patients would be considered to have moderate disease severity. These values were taken from a widely used and well established criterion for summarizing disease stages in glaucoma (Hodapp et al., 1993; Mills et al., 2006).

Three other vision tests were performed on the day of the experiment. Corrected binocular visual acuity (BVA) was measured using an Early Treatment Diabetic Retinopathy Study (ETDRS) chart. All participants recorded BVA of at least 0.18 logMAR (Snellen equivalent of 6/9). We chose to restrict the study to patients with preserved VA to allow for the impact of glaucoma to be better isolated. Contrast sensitivity (CS) was measured in log units with a Pelli-Robson chart. The Oculus C-Quant straylight meter (Oculus GmbH, Wetzlar, Germany) was used to measure abnormal light scattering in the eye media, in order to eliminate significant media opacity and other lens type artifacts as confounding ocular conditions; all participants were required to be within “normal limits” for this test.

All participants were examined with a modified version of the Middlesex Elderly Assessment of Mental State (MEAMS, Pearson, London, UK), a psychometric test designed to detect gross impairment of specific cognitive skills such as memory and object recognition in an elderly population. Each section of the MEAMS is scored independently with lower scores indicating a more significant cognitive impairment. The use of MEAMS for screening of impairments has been validated by a number of research studies (Morris et al., 2000). All participants passed the MEAMS test. Individual scores (percent) were also recorded to compare patients with controls.

### Main experiment

Participants viewed three separate unmodified TV and film clips with sound on a 54 cm monitor (Iiyama Vision Master PRO 514, Iiyama Corporation, Tokyo, Japan) at a resolution of 1600 by 1200 pixels (refresh rate 100 Hz). One clip was an excerpt from an entertainment program (309 sec; *Dads Army*, BBC Television) which covered the full screen (subtending a half-angle of 20.3° by 14.9°). The other two clips were taken from a feature film (200 sec; *The History Boys*, 20th Century Fox) and a sports program (436 sec; *2010 Vancouver Winter Olympics Men's Ski Cross*, BBC Television); both these clips were recorded at a 16:9 ratio, therefore they contained black rectangles at the top and bottom of the screen (subtended a half-angle of 17.3° by 10.6°). Participants were positioned, using a chin rest, at a viewing distance of 60 cm. The volume was kept at the same level throughout all the trials and the films were in color. All participants wore trial frames with a refractive correction suitable for the viewing distance of 60 cm to ensure that any obstruction to the field of view caused by spectacle frames would be equivalent for everyone.

Monocular eye movements were recorded at a temporal resolution of 1000 HZ during the task using an SR Research EyeLink 1000 (SR Research Ltd., Ontario, Canada). A purpose-written application developed in C++ using Microsoft DirectShow was used to display the video and link with the SR Research EyeLink API. The eye giving the best quality pupil detection and corneal reflection was chosen for tracking. The EyeLink proprietary algorithm was used to calibrate and verify the participants' point of regard in relation to the correct location on the display. After measuring fixation the default calibration technique monitors eye movement to a stimulus presented at nine points on the monitor. This process is repeated giving a measure of the validation of the calibration. This process only took a few minutes at most. Calibration accuracy flagged by the system to be of a “good” level was a prerequisite before each trial. A drift correction was also performed before each of the three films was displayed, and in the case where a large drift (greater than 5°) was detected, a recalibration performed. The films were shown in the same order for all participants.

### Eye movement and data analysis

The Eyelink 1000 gives average eye position accuracy of better than 0.5° and uses velocity and acceleration thresholds of 30°/s and 8000°/s^2^, respectively to identify saccades. This simple definition means smooth pursuits are excluded. Since the recordings were done over a relatively long uninterrupted period of time then it was not unusual for the eye tracker to lose the position of the point of regard because of blinks and loss of pupil position. In these instances the data is not useful. An automated filtering technique was applied to the collected data. A sliding window, passing over the entire temporal eye movement trace associated with each film for each person, counts the number of saccades made per second. The percentage of 1 sec regions containing one or more saccades is delineated per video clips per person and labeled as “good” data. Any clip that contains less than 60% of these regions of “good” data is excluded from the analysis. This filtering was only used to exclude whole film clips. If a film clip was included then all the available measured saccades made whilst viewing it were used.

**Figure 1:**
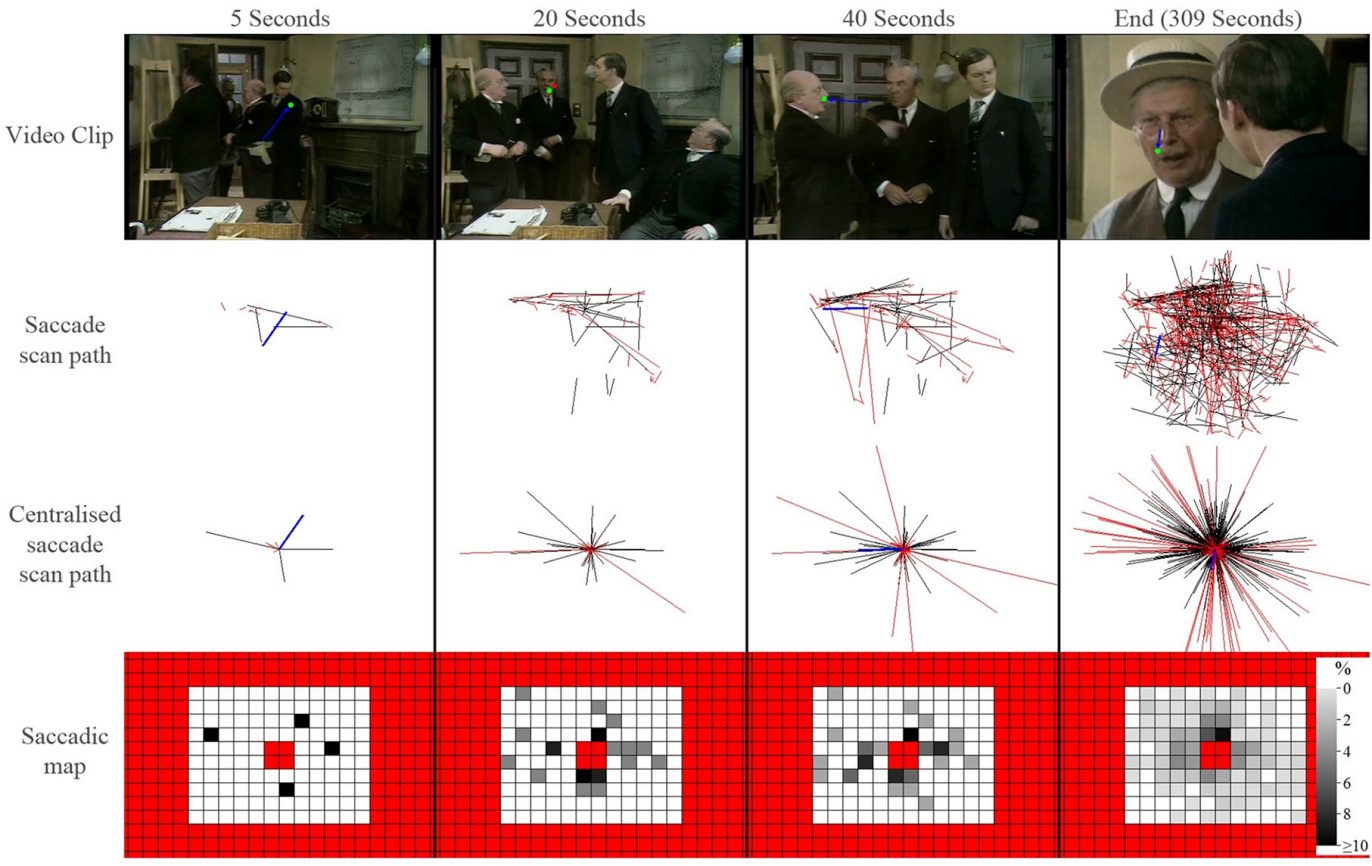
**A schematic illustrating how saccade maps are constructed from one control participant viewing one video clip**. The top row shows frames from a video clip at a set time (green symbol shows a fixation and blue line represents the preceding saccade). The *saccade scan path* shows all saccades that have occurred up until that point in the video. The *centralized scan path* shows how the saccade is treated as vector starting at the origin (0, 0) on a Cartesian grid. The red lines highlight saccades excluded for being either too large or too small. A saccade heat map is created from the centralized saccade scan path: the darker each location in the map, the larger the percentage of saccades that ended in that region of the visual field (red regions represent excluded locations). Each square is 2°.

Next our software application builds a scanpath of saccades and fixations for each person viewing each clip. From this a saccade map is constructed on a grid of size 12° by 10° half angle subdivided into 2° regions. This grid is populated with the end position of every single saccade after it is assumed to be a vector starting at the origin (0, 0) on a Cartesian grid. Saccades with end points falling outside this region are excluded. In addition, the central 4 regions are excluded from the map thus removing indeterminable saccades of small amplitude. The frequency distribution of the saccade endpoints across the entire map is then recorded. This methodology is illustrated with the schematic shown in Figure 1 and in a movie given in Supplementary Material. For the next stage of the analysis we have a matrix of 116 values (10 × 20 with 4 central excluded values) for each video clip for each person.

We first use kernel principal component analysis (KPCA) to extract classifiable features from the saccade maps. KPCA is widely used in pattern recognition and image processing problems (Scholkopf et al., 1999; Kwang In Kim et al., 2002; Jian Yang et al., 2005). In short, KPCA is similar to PCA but it handles non-linearities in the data by implicitly transforming data into high dimensional feature space via the kernel function and then performing a linear analysis in that space. The technique extracts features in an unsupervised fashion meaning, from a practical point of view, that none of the saccade maps are labeled as a case or a control. The feature space in KPCA is defined by “kernels” that quantifies the “distance” measure between every pair of participants. Each saccade map is serialized to a 116 point vector and the difference between participants on one video trial is defined as the Euclidean distance between the two corresponding vectors.

The kernel *k_ij_* between two participants *i* and *j* is then defined as a non-normalized Gaussian distribution (1) where *meanDiff* and *maxDiff* are the mean and maximum difference of all video trials between the two participants:

(1) $$k_{ij}=e^{-\frac{1}{2}\frac{{\left(meanDiff+maxDiff\right)}^{2}}{0.2^{2}}}$$

The kernel *k_ij_* forms the KPCA feature Gram matrix **K** which is then normalized and decomposed into principal eigenvectors, the importance of these is evaluated by ranking the corresponding eigenvalues. The features for each person in our study are then calculated as the projection onto these principal axes. Crucially we then hypothesize that these “mathematical” features carry characteristics that will allow us to efficiently separate the patients from the controls on these data alone. In order to do this, significant features with the highest eigenvalues are input into a Naïve Bayes linear classification algorithm. The classifier is trained and tested by a 10 fold cross validation on all the data, repeated 100 times. In particular, with each iteration of the cross validation, 90% of the participants are randomly sampled to train a Naïve Bayes model; the diagnostic performance of this model is then tested on the remaining 10% participants. The average sensitivity of the technique is then estimated (with 95% confidence intervals) at fixed specificity using all iterations for the entire sample of the data. These values are used to construct a Receiver Operating Characteristic (ROC) curve summarizing the classification potential of the methodology.

The analytical methods described were implemented in purpose written programs using MATLAB R2013a (MathWorks Inc., Natick, MA).

## Results

**Figure 2:**
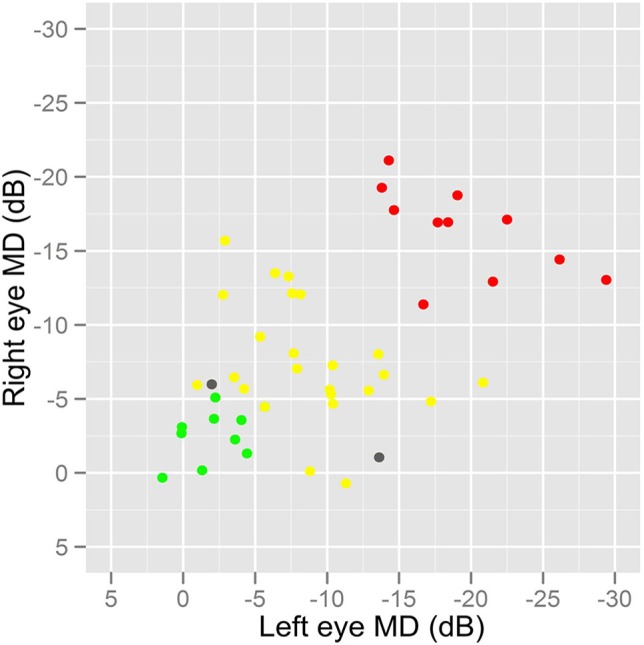
**A graph showing the distribution of disease severity in the patients**. HFA MD values were used as a measure of overall glaucomatous disease severity in the patient group. Patients with MD better than −6 dB in both eyes were classified as having early glaucomatous damage (green) whilst those with MD worse than −12 dB in both eyes were considered to have advanced disease (red). All other patients would be considered to have moderate disease severity (yellow). Two patients, excluded because of sufficient eye tracking data, are shown as gray symbols.

Seventy-eight people were recruited and took part in the study. We failed to acquire sufficient eye tracking data in two individuals (both patients). Therefore, our study sample comprised 44 patients and 32 healthy people (controls) with mean age of 69 (standard deviation [SD]: 8) and 68 (SD: 9) years, respectively. These means were not significantly different (two sample *t*-test; *P* = 0.58) and the distribution of the ages were also equivalent (*F*-test of variances; *P* = 0.35) meaning the groups represent age-similar populations. Twenty-two of the patients (50%) and 17 controls (53%) were female. Glaucoma disease severity in the patient group, as described by HFA MD in both eyes, is shown in Figure 2; 9 (20%) and 11 (25%) of the patients had early and advanced glaucomatous disease, respectively. Median (interquartile range) right eye and left eye MD was −7 (−13 to −5) dB and df−9 (−15 to −4) dB, respectively. Summary statistics for the other vision and supplementary tests are shown in Table 1. As expected CS differed between groups. The difference between group BVA was statistically significant (*P* = 0.002) but the actual size of the average difference was clinically small (95% CI for the mean difference of 0.03–0.13), representing about 4 letters on the chart, reflecting our minimum acuity inclusion criterion.

**Table 1:** **Group summary statistics (mean [SD]) and comparison (two-sample *t*-test) for ETDRS corrected binocular LogMAR visual acuity (BVA), Pelli-Robson contrast sensitivity and MEAMS**.

	**Patients *n* = 44**	**Controls *n* = 32**	***p*-value**
BVA (LogMAR)	0.02 (0.13)	−0.06 (0.09)	0.002
CS (Log)	1.84 (0.18)	1.95 (0.00)	<0.001
MEAMS (%)	97.9 (3.7)	98.1 (3.9)	0.82

**Figure 3:**
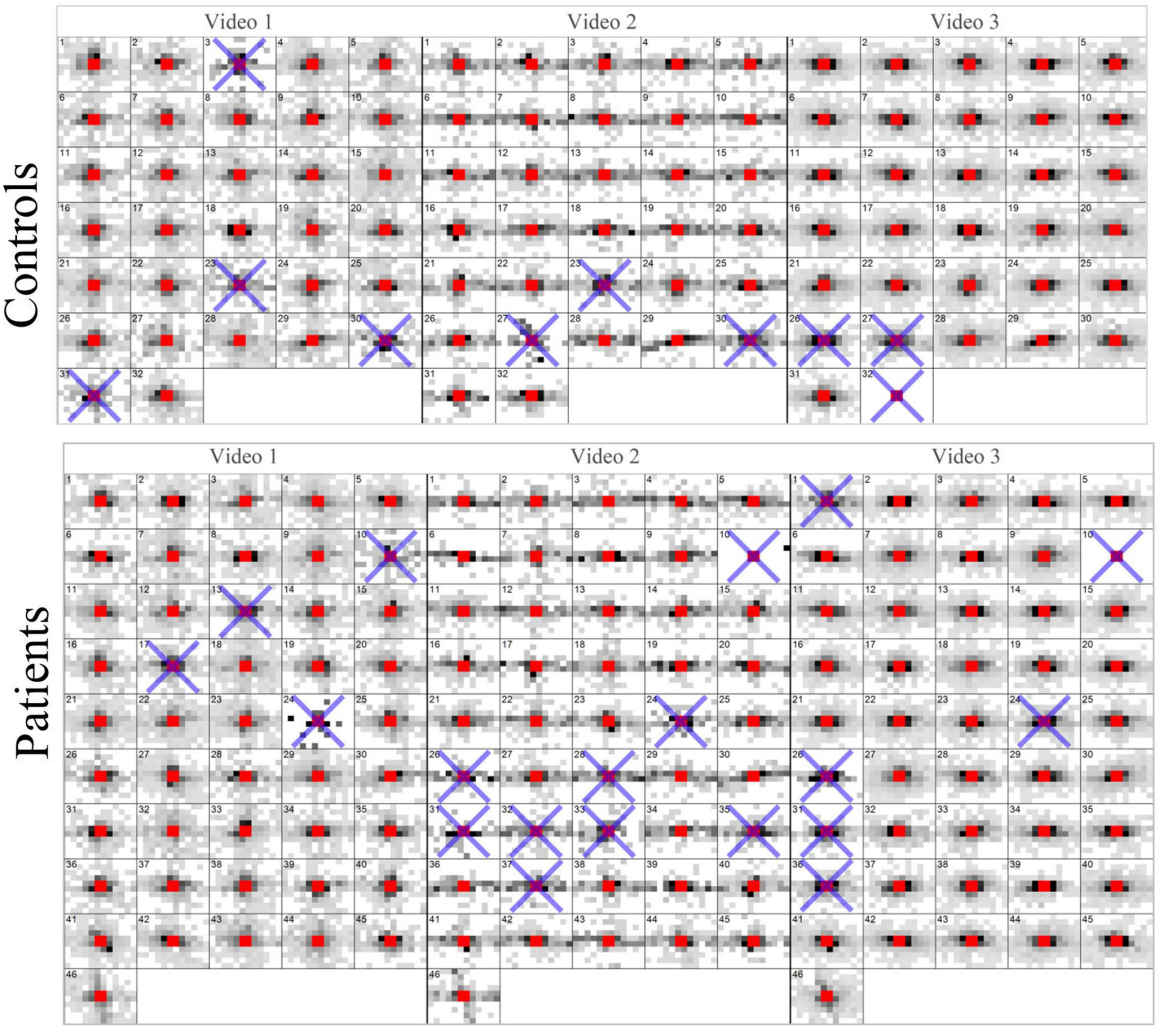
**Saccade maps for every video clip for every participant**. A blue cross indicates a trial (video) that did not have enough valid eye movement data according to the filtering process. These maps represent the entire data processed by the KPCA.

Saccade maps for every film clip for every participant are given in Figure 3. In total 205 out of 234 (88%) film clips were wholly included in the analysis. There was no statistically significant difference (Chi-Squared Test; *P* = 0.43) between the average inclusion rates for the controls (92%) and patients (86%).

**Figure 4:**
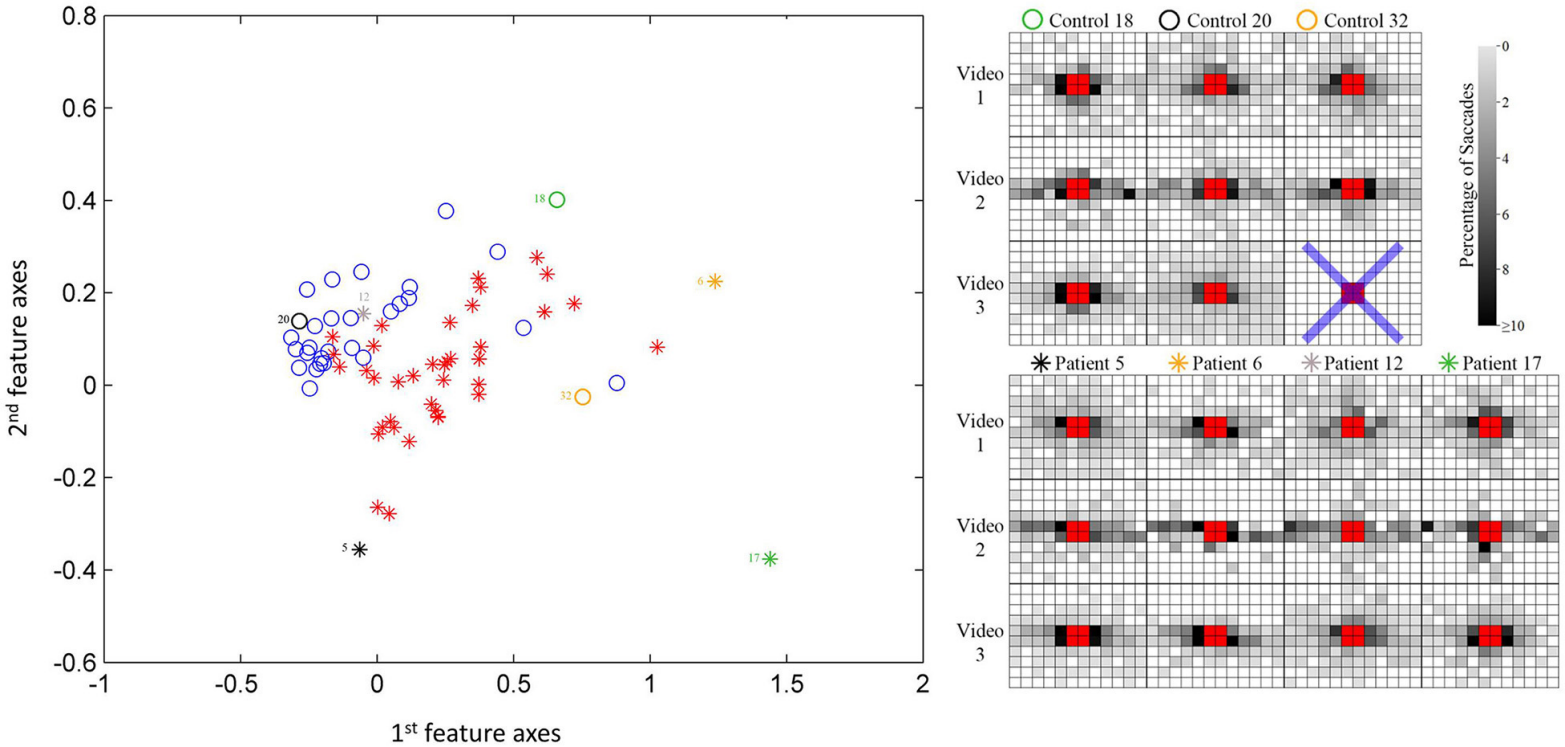
**Scatterplot of participant data on the first two most significant feature axes from the KPCA; this provides a visualization only of the reasonable separation between patients and controls**. Note the classifier is blind to the diagnosis. Example saccade maps (right) from three controls and four patients for all three videos in this study. The blue cross represents a trial that does not have enough valid eye movement data according to the filtering process. The symbols correspond to the location of the subject in the scatterplot.

**Figure 5:**
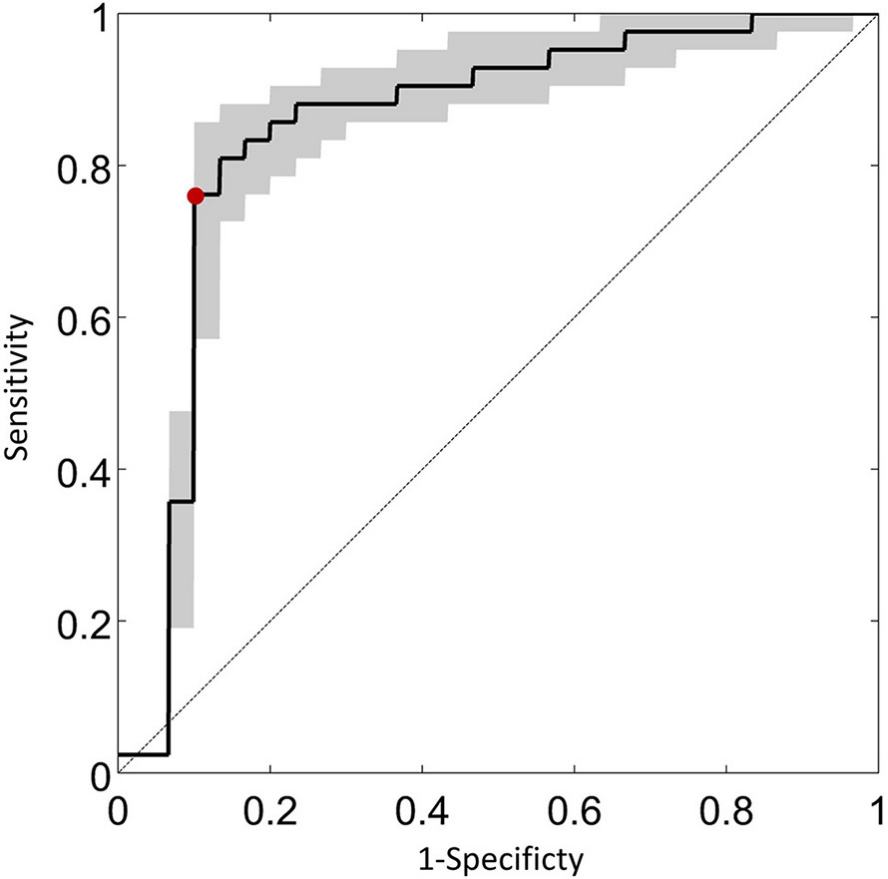
**An ROC curve summarizing the classification potential of the methodology**. The average sensitivity of the technique estimated (with 95% confidence intervals) at fixed specificity using all iterations for the entire sample of the data is shown on the vertical axis. The red symbol illustrates that the technique has a sensitivity of 76% (95% confidence interval of 58–86%) at 90% specificity. The diagonal line is the classification by chance.

An illustration of the results from the feature extraction, as applied to the saccade maps, is given in Figure 4. Here the two most significant features (eigenvectors, in arbitrary units) are plotted against each other. This visualization hints at the good separation achieved by considering the saccade maps alone when the data is reduced to just two feature axes. KPCA revealed that the five feature axes with the largest eigenvalues accounts for 35% variance in the data. These five feature axes were then used in the Naïve Bayes linear classification algorithm. An ROC curve summarizing the “diagnostic precision” of the classifier is shown in Figure 5. The area under the ROC curve is 0.85 (95% confidence interval of 0.82–0.87). One point is highlighted on the ROC curve since this illustrates that the technique has a sensitivity (hit rate) of 76% (95% confidence interval of 58–86%) at 90% specificity.

## Discussion

A whole series of visual and neurological processes coalesce in order to allocate gaze efficiently. It therefore seems reasonable that gaze patterns might be inhibited or be altered by visual and neurological disorders. This simple notion underpins our driving hypothesis that scanpaths from gaze patterns might be idiosyncratic of neurodegenerative conditions. It might be that these patterns can only be realized after examining an extensive period of recorded eye movements, but this is now possible with modern eye tracking equipment and statistical methods that can interpret the mass of scanpath data that is yielded by them. In this case-control study we chose patients with one particular neurodegenerative eye disease. Gaze patterns were examined simply whilst viewing everyday TV type films in a quite uncontrolled fashion. The results from this study demonstrate proof of principle for the effectiveness of our approach. Using novel methodology we show that features extracted from extensive maps of saccades made whilst watching TV clips can be quantified to correctly differentiate a group of patients with a clinical diagnosis of glaucoma from a group of age-similar healthy people. For example, at a relatively high specificity (90%) we classified a patient in our sample with sensitivity of 76% by using the saccade maps alone.

Some context for this estimate of “diagnostic accuracy” is needed. There are three well-established index tests used to detect glaucoma: intraocular pressure (IOP) measurements, visual field tests and clinical assessments of the optic nerve. A systematic review of the clinical effectiveness of detecting glaucoma reported an enormous range for the estimates of diagnostic performance in these tests (Burr et al., 2007; Mowatt et al., 2008). The pooled estimates of the sensitivity of the clinically used tests in detecting COAG ranged from as low as 42–92%, whilst specificity ranged from 75 to 95%. Elevated IOP is only a risk factor for disease but tonometry, the instrumentation used to assess high IOP, is routinely used in primary care and case finding. The lower estimates for diagnostic accuracy (~ 40–50%) were for IOP measures. Unsurprisingly those index tests reported to have better performance tended to be perimetric tests sharing direct attributes with the reference standard. Methods for assessing the ONH, either by direct observation by expert ophthalmologist or an imaging device fare no better in detecting glaucoma, yielding only modest accuracy when used in isolation. In one well reported study, even expert ophthalmologists were shown to only classify ONH photographs moderately well for detecting glaucoma with diagnostic accuracy similar to what we report in our study (Reus et al., 2010).

Our findings need to be discussed in the context of current research. A catalog of research, extensively reviewed elsewhere, suggests that laboratory recordings of eye movements can provide valuable information about neurodegenerative disease, and hold promise as biomarkers for characterizing the efficacy of neuroprotective and neurorestorative therapies (Anderson and MacAskill, 2013). At the same time research findings about the nature and consistency of gaze patterns shown by groups of individuals as they view scenes or films are somewhat mixed (Borji and Itti, 2014). On the one hand, studies have shown that individuals within a group tend to produce similar eye movements for the same movie of natural scenes; a finding thought to be influenced by scene driven saliency effects whereby certain properties of the image cause individuals to produce a certain type of eye movement (Itti, 2006; Cristino and Baddeley, 2009; Tatler et al., 2011). Conversely, some studies have reported high levels of variability of eye movements between subjects in response to viewing scenes, which could be indicative of idiosyncratic viewing patterns within an individual (Andrews and Coppola, 1999; Castelhano and Henderson, 2008), whilst others, for example, have shown eye movements to natural images vary enormously as a function of personality (Mercer Moss et al., 2012). More recently other workers have pursued the effect of age in processing video (Kirkorian et al., 2012). Others did not find evidence for idiosyncratic viewing patterns of the same subject across different movies (Dorr et al., 2010). Our study is most closely aligned with the attempts made to classify patients with attention deficit hyperactivity disorder, fetal alcohol spectrum disorder and Parkinson's disease using natural viewing eye movements by Tseng et al. (2013). A novel computational model of visual attention based on saliency properties of each frame of specifically constructed scene-shuffled videos was used as a benchmark to predict gaze. By using a case-control study, similar in design to ours, the investigators managed to classify a small group of patients with acceptable levels of diagnostic accuracy. The investigators also used machine learning methods to analyses the eye movement data and to classify the differences between predicted gaze and actual gaze in the individuals (Tseng et al., 2013). Our work differs because we did not build a model predicting where people should look. Neither did we specifically select dynamic content to be used. The novelty of our hypothesis rested on the principal of demonstrating that patients could be separated from controls where the dynamic content being viewed was not extensively controlled. Our experimental effect was surprisingly large given this condition.

The results from this study hint at important clinical applications and we speculate on these briefly now. Potential tests of age-related neurological conditions, like glaucoma, based on the concept formulated in this study would require little patient action beyond the passive viewing of movies. Such a procedure would have the potential to provide a continuous assessment of changes either as the disease developed, or during treatment, within a more realistic visual environment. Moreover, eye tracking will likely become more affordable, practical, and robust in the near future, driven not by scientific research but by demands of computer gaming, mobile technology, and developments in human computer interaction. Our contention is that anomalies in eye movements tracked during viewing of naturalistic stimuli have the potential to be developed into a rigorous test that could be incorporated into an everyday activity, like something as simple as watching a movie.

The experimental design of our study had several strengths. The sampling was done carefully to ensure that the cases had the same age, sex, and general health profile as the controls. All participants passed the MEAMS test for cognitive ability and average scores from this test did not differ between the two groups. Our experimental design tried to eliminate other visual factors affecting the results. For example, we chose to restrict the study to patients with preserved visual acuity and healthy optical media (assessed by straylight measures) to allow for the contribution of functional loss due to glaucoma to be better isolated. In addition, the patients in this study had a range of disease severity (Figure 2) adding to the strength of our findings. (Tests with diagnostic promise become more sensitive as the disease becomes more severe; a study including participants with advanced disease only would by default report better sensitivity.) Of course, at this stage, it is very important to acknowledge results from this preliminary case-control report do not even remotely suggest that our method would translate into clinically significant gains in the diagnostic precision of the disease; this must be the subject of a study that must follow appropriate standards (Bossuyt et al., 2003).

Our study had other notable novel attributes. For example, the majority of eye movement studies reported in the literature have been done on trained observers or young healthy volunteers: our study clearly demonstrates that it is it is possible to collect extensive eye movement data on elderly people; more than one quarter of our participants were older than 75 years. Moreover, we took advantage of modern statistical methods (KPCA) to assess the high dimensional data yielded by the eye tracking. These methods are unsupervised and “learn” the discriminatory features without training on data that is labeled as “case” or “control.”

There are several limitations to our study. This proof of concept study was designed to develop our method and test it on the same material. It is well established that if modeling and testing is done on the same data then model estimates of effects will be overly optimistic. A far better experiment would have used one sample of participants to develop the classifier and another sample for testing. Our sample size was relatively small, although large enough to demonstrate an effect. All the testing was done on the same experimental set up; the repeatability of these results on another experimental set up is unknown. The study included no tests for attention deficits and so we cannot be sure that the two groups would have had the same level of attention. Furthermore, assessment for cognition was based on a modified screening test only. Therefore, we cannot be certain that the two groups would be the same had a more through cognitive examination been carried out. Two patients were excluded because we could not extract meaningful scanpath data from them, and this would be important if clinically meaningful estimates sensitivity and specificity were being reported. Moreover, our results and the paucity of data offer no real hint about what particular characteristics of the saccade maps are suggestive of abnormality and this awaits further study. Moreover, only patients with glaucoma have been considered and we have no idea if these results will translate to other age-related neurodegenerative conditions. Furthermore, the pathogenesis of glaucoma shares many features with other chronic age-related neurodegenerative disease but it is not typically or primarily classified as such. The site of damage histologically appears to be at the level of the ONH and is thought to be due to the interaction of IOP, cerebrospinal fluid pressure, ONH blood flow and changes in lamina cribrosa anatomy with retinal ganglion cell changes being secondary.

In conclusion we have shown scanpaths of eye movements recorded whilst people freely watch TV type films can be processed into maps that contain a signature of age-related neurological disease. In this *proof of principle* study we have demonstrated that a group of patients with glaucoma can be reasonably well separated from a group of healthy peers by considering these eye movement signatures alone. Future studies will consider larger groups of patients and other age-related neurological disorders.

### Conflict of interest statement

The authors declare that the research was conducted in the absence of any commercial or financial relationships that could be construed as a potential conflict of interest.
